# Adjuvants Enhancing Cross-Presentation by Dendritic Cells: The Key to More Effective Vaccines?

**DOI:** 10.3389/fimmu.2018.02874

**Published:** 2018-12-13

**Authors:** Nataschja I. Ho, Lisa G. M. Huis in 't Veld, Tonke K. Raaijmakers, Gosse J. Adema

**Affiliations:** ^1^Radiotherapy and OncoImmunology Laboratory, Department of Radiation Oncology, Radboud Institute for Molecular Life Sciences, Radboud University Medical Center, Nijmegen, Netherlands; ^2^Department of Anesthesiology, Pain and Palliative Medicine, Radboud University Medical Center, Nijmegen, Netherlands

**Keywords:** adjuvants, dendritic cell, cross-presentation, aluminum, saponin, TLR, vaccine

## Abstract

Over the last decades, vaccine development has advanced significantly in pursuing higher safety with less side effects. However, this is often accompanied by a reduction in vaccine immunogenicity and an increased dependency on adjuvants to enhance vaccine potency. Especially for diseases like cancer, it is important that therapeutic vaccines contain adjuvants that promote strong T cell responses. An important mode of action for such adjuvants is to prolong antigen exposure to dendritic cells (DCs) and to induce their maturation. These mature DCs are extremely effective in the activation of antigen-specific T cells, which is a pre-requisite for induction of potent and long-lasting cellular immunity. For the activation of CD8^+^ cytotoxic T cell responses, however, the exogenous vaccine antigens need to gain access to the endogenous MHCI presentation pathway of DCs, a process referred to as antigen cross-presentation. In this review, we will focus on recent insights in clinically relevant vaccine adjuvants that impact DC cross-presentation efficiency, including aluminum-based nanoparticles, saponin-based adjuvants, and Toll-like receptor ligands. Furthermore, we will discuss the importance of adjuvant combinations and highlight new developments in cancer vaccines. Understanding the mode of action of adjuvants in general and on antigen cross-presentation in DCs in particular will be important for the design of novel adjuvants as part of vaccines able to induce strong cellular immunity.

## Introduction

Since the development of the first successful vaccine by Edward Jenner in 1796 against smallpox, a lot of research has been done on the development of vaccines against other diseases. Current vaccines against infectious agents can be divided into live attenuated vaccines (where their virulent properties are weakened, e.g., yellow fever, measles), subunit vaccines (containing a fragment of the pathogen, e.g., Hepatitis B), toxoid vaccines (with inactivated toxic compounds, e.g., tetanus, diphtheria), and conjugated vaccines (linking polysaccharide coats to protein, e.g., *Haemophilus influenzae* type B) (1). While especially prophylactic vaccines against infectious diseases have been developed successfully and are clinically applied, development of therapeutic vaccines against persistent infections or cancer is lagging behind. For the development of new vaccines many aspects should be taken into consideration such as the nature of the antigenic material, the type of immune memory responses that needs to be induced, but also the administration and delivery routes, which might reduce the risk of side effects. Next generation vaccines like subunit vaccines for infectious diseases mostly aim for higher safety with less side effects, which is often detrimental for their immunogenicity. Therefore, adjuvants are usually required to enhance vaccine potency. Similarly, tumor neoantigen vaccines are devoid of immune activation potential and are fully dependent on strong adjuvants to induce protective immune responses. Adjuvants generally act by activating innate and adaptive immune responses, but can also function to create an antigen depot, slowly releasing the antigen for prolonged presentation and stimulation of the immune system (2). One of the first licensed carrier-adjuvants was alum, an inorganic adjuvant widely used in vaccines against e.g., hepatitis B virus, human papillomavirus, and diphtheria. Like most of the early adjuvants, they were mainly aimed at inducing protective antibody responses and hence strongly Th2 biased immunity. The discovery of microbe sensing pattern recognition receptors (PRRs), such as Toll-like receptors (TLRs) and nucleotide-binding oligomerization domain (NOD)-like receptors, has boosted research into vaccine adjuvants aiming to induce cellular immune responses that are essential to fight intracellular pathogens and cancer cells. Interaction of PRR with their corresponding ligands potentiate and shape the adaptive immune responses (3). Since then, several types of immune potentiating adjuvants (e.g., TLR agonists and saponin QS-21) have been licensed and used in the clinic against various diseases (Table 1).

**Table 1:** Clinically approved adjuvants.

**Adjuvant**	**Description**	**Proposed immune mechanism**	**Clinical application**
Aluminum salts	Hydroxide, phosphate, alum	Activation of NLRP3 inflammasome and caspase-1 in DCs, induces Th2 response (4, 5).	HBV, HPV, diphtheria, and tetanus
AS01	Liposome (containing MPL and QS-21)	Activates APCs expressing TLR4, stimulates cytokine and co-stimulatory molecules production, promotes antigen-specific antibody responses and stimulates CD8^+^ T cells (6).	Malaria, Herpes Zoster
AS02	Oil-in-water emulsion (containing MPL and QS-21)	Antigen specific CD8^+^ and CD4^+^ T cell responses and antibody responses (7)	Malaria
AS03	Oil-in-water emulsion (containing squalene, polysorbate 80 and α-tocopherol)	NF-κB activation, production of cytokines and chemokines in muscle and draining LN, provoke migration of monocytes, DCs and granulocytes into draining LN, enhancing CD4^+^ T cell immune responses (8).	Pandemic influenza
AS04	MPL formulated in aluminum salt	Activates TLR4 on DCs, induction of cytokines and antigen specific T cell activation (9).	HBV, HPV
MF59	Oil-in-water emulsion	Rapid influx of CD11b^+^ cells, upregulation of inflammatory cytokines and chemokines, recruitment of APCs (10).	Seasonal and pandemic influenza
Virosomes	Lipid vesicle containing inactivated viral proteins	Virosomal-adjuvanted influenza vaccine (Inflexal®V) increases antibody titer (11).	Influenza, Hepatitis A

*NLRP3, nucleotide binding domain-like receptor protein 3; DCs, dendritic cells; HBV, Hepatitis B virus; HPV, human papillomavirus; MPL, monophosphoryl lipid; LN, lymph node*.

Each adjuvant has a unique immunological signature that can be used in highly different types of diseases. Choosing the right adjuvant to combine with the best target antigen for a given disease is a challenging task (12). Next generation vaccine adjuvants are now mostly designed to contain both the function of a carrier and a potent immune response inducer to boost the efficacy of the vaccine. Although many prophylactic vaccines rely on neutralizing antibody responses, especially diseases such as cancer, HIV, tuberculosis, and malaria are in need of a vaccine eliciting strong T cell responses (13–17). As a consequence, many studies investigated the potency of next generation adjuvants for their capacity to induce antigen specific CD8^+^ and CD4^+^ T cell responses. An important characteristic of adjuvants able to induce cellular immunity is the efficient delivery of the target antigen into professional antigen presenting dendritic cells (DCs) and its potency in activating these DCs. In general, DC maturation enhances their antigen presentation capacity and ability to activate T cells and is a prerequisite for induction of potent and long-lasting immunity. One of the best studied DC maturation stimuli are TLR ligands, including poly(I:C), LPS, CpG, R848, and Pam_3_CSK_4_, which can activate DCs to upregulate co-stimulatory molecules such as CD40, CD80, and CD86 (18). TLRs can be expressed extracellularly (TLRs 1, 2, 4, 5, and 6) and intracellularly (TLRs 3, 7, 8, and 9) (3). All TLRs, except TLR3, utilize the adaptor molecule MyD88 to trigger activation of TGF-β Activated Kinase 1 which activates MAPK and NF-κB signaling resulting in TNF-α, IL-12, and IL-6 production (19, 20). Intracellular TLRs, which are mostly found in endosomes, require internalized ligands such as nucleic acids to activate downstream signaling. Currently, only the TLR4 agonist monophosphoryl lipd (MPL), a non-toxic LPS-derived TLR4 ligand, is approved for human applications (Table 1). Other TLR ligands showed effective tumor immunity in animal models or clinical trials (21–23).

Alternative pathways for DC maturation include intracellular receptors, such as Nucleotide binding domain-Like Receptor Protein 3 (NLRP3), which forms a caspase-1 activating complex (inflammasome) together with Cardinal and apoptosis-associated speck-like protein containing a caspase recruitment domain (24). This pathway results in cleavage and release of the pro-inflammatory cytokines IL-1β, IL-18, and IL-33 (25). A very important characteristic of adjuvants that has received much less attention is their ability to induce presentation of exogenous antigens not only in MHCII to CD4^+^ T cells but also in MHCI to CD8^+^ T cells. This latter process is essential for efficient CD8^+^ T cell priming and is called antigen cross-presentation. In this review, we will focus on recent insights in clinically relevant adjuvants that impact DC cross-presentation. Understanding DC cross-presentation will be important to design novel adjuvants able to induce strong cellular immunity for future vaccine development.

## Molecular Mechanisms of Dendritic Cell Cross-Presentation

Dendritic cells are the professional APCs of our immune system that are key in linking innate and adaptive immunity. DCs are especially known for their ability to cross-present, as they process and present exogenous antigens on MHCI molecules much more efficiently than other phagocytes. The efficiency of CD8^+^ T cell priming called cross-priming by DCs is dependent on both antigen cross-presentation efficiency (number of a given MHCI/peptide complex on the cell surface) and the level of DC maturation (expression levels of co-stimulatory molecules and cytokines). It has been reported that cross-presentation is important for inducing T cell responses specific for tumor antigens and infectious diseases (26–28). How exogenous antigens are processed in DCs and presented on MHCI to CD8^+^ T cells is still not fully understood. Two main pathways of antigen cross-presentation in DCs have been proposed: the cytosolic pathway and the vacuolar pathway. In the cytosolic pathway, exogenous antigens or protein fragments derived from it are transported from endosomal vesicles into the cytosol where they are degraded by the proteasome. The trimmed peptides are then transported by the transporter associated with antigen processing (TAP) to the endoplasmic reticulum (ER) where they are loaded on MHCI molecules (29–31). However, there have been suggestions that the protein fragments can be transported back into endocytic compartments and trimmed by insulin-regulated aminopeptidase (IRAP) and loaded on MHCI (32). In the vacuolar pathway antigens are degraded by proteases in endo/lysosomal compartments and directly loaded on MHCI molecules (33, 34). A comprehensive overview of these and alternative cross-presentation pathways in DCs has recently been reviewed (35).

How antigens are transported from the endosomes to the cytosol is still under debate. Extensive studies in murine models identified the ER-associated degradation (ERAD) member, Sec61, as a possible translocator for antigen from the endosomes into the cytosol. Applying a Sec61-specific intracellular antibody, Zehner et al. showed that they could trap Sec61 in the ER and prevent its transport toward endosomes, thereby blocking antigen translocation and cross-presentation (36). However, a more recent study using mycolactone, which binds specifically to Sec61α, showed severe inhibition of protein import into the ER but no inhibition of ERAD or protein export from endocytic compartments (37). Although, both studies showed inhibition of DC cross-presentation upon blocking of Sec61, it seems that Sec61 plays a more dominant role in inhibiting protein translocation into the ER and altering antigen cross-presentation at a different level than antigen export to the cytosol.

Another ongoing debate is how ER proteins are translocated to endosomes in DCs for efficient cross-presentation. The group of Amigorena proposed that recruitment of ER and ER-Golgi intermediate compartment (ERGIC) components to phagosomes is mediated by the ER-resident SNARE Sec22b (38). Silencing of Sec22b uncovered that phagosome-lysosome interactions were delayed, thereby limiting proteolysis and preserving antigenic fragments for cross-presentation, which was recently also confirmed in conditional Sec22b-knockout DCs (39). Conflicting results were found using similar Sec22b-knockout DCs (40) and based on a review of both studies with respect to technical differences, a role for Sec22b as well as for unidentified new regulators of cross-presentation was suggested (41). Although Sec22b seems to regulate antigen cross-presentation in the vacuolar pathway, it is not ruled out that it can play a role in the cytosolic pathway.

Two recent studies report on regulation of antigen cross-presentation in DCs by stromal interaction molecule 1 (STIM1), a calcium sensor that conveys the calcium content of the ER to store-operated channels of a cell (42, 43). Nunes-Hasler and colleagues showed that STIM1 can promote the contact sites between the ER and phagosomes (42). This induces Ca^2+^ signaling and thereby the migration and fusion of phagosomes with endosomes or lysosomes to enable efficient cross-presentation in DCs. In a companion study it was shown that the ER membrane protein uncoordinated 93 homolog B1 (UCN93B1) interacts with STIM1 and can control cross-presentation in DCs (43). Ablation of UCN93B1 impairs phago-lysosomal fusion, proteolytic activity, and antigen export to the cytosol, resulting in a decrease of antigen degradation and cross-presentation. Others showed that antigen transportation into the cytosol is enhanced by NADPH-oxidase complex (NOX2) and reactive oxygen species (ROS) production in the endosomes (44). Reactive oxygen species causes lipid peroxidation, which disrupts the endosomal membrane, resulting in antigen leakage from endosomes. Furthermore, it has been shown that NOX2 can be recruited to the endosomes to induce alkalization upon ROS release (45). This will cause an increase of endosomal pH thereby preventing rapid antigen degradation, resulting in enhanced antigen cross-presentation. The group of Guermonprez suggested that lipid bodies (LBs) are involved in DC cross-presentation (46). They showed that the Immunity-related GTPase family member 3 (Irgm3) controls accumulation of LBs induced by cell activation stimuli including INF-γ and Poly(I:C). LBs are organelles composed of a central core of cholesteryl esters and triglycerides that are surrounded by a single layer of phospholipids also containing LB proteins (47). The Irgm3 protein is localized in the ER and in LBs where it interacts with the LB coat protein adipose differentiation-related protein (ADRP). Mice deficient in either Irgm3 or ADRP showed defects in LB formation and impaired cross-presentation in DCs. Further research is needed to understand how LBs control antigen cross-presentation by DCs and to determine the molecular pathways that control the involvement of LBs.

## Antigen Cross-Presentation and DC Subsets

An important aspect to take into account when choosing an adjuvant to induce DC cross-presentation is the type of DC that will be affected. Intensive research has shown that there are many DC subsets present in mice as well in human, with still room for newly unidentified subsets. Murine DCs in secondary lymphoid organs can be divided roughly into conventional DCs (cDCs) and plasmacytoid DCs (pDCs). cDCs can be further divided into cDC1 (CD8α^+^ and CD103^+^) and cDC2 (CD8α^−^, CD11b^+^, and CD172a^+^) DCs (48). The development of CD8α^+^ DCs is regulated by the transcription factors including inhibitor of DCN binding 2 (Id2), interferon regulatory factor (IRF) 8, basic leucine zipper ATF-like 3 transcription factor (BATF3), and the nuclear factor interleukin 3 regulated (NFIL3) (49). The development of CD8α^−^ DCs is orchestrated by the transcription factors including RelB, NOTCH2, RBP-J, IRF2, and IRF4. Deletion of either of these genes can lead to developmental defects of the DC subsets. Mice in which a given DC subset has been selectively depleted, e.g., Batf3^−/−^ mice or zinc finger transcription factor knockout studies, have provided important insight in the functional role of DC subsets in antigen presentation (50, 51). However, the interpretation of the data in these mice regarding cross-presentation is not always straightforward due to incomplete depletion, depletion associated side effects, and DC cross-talk. In general, CD8α^+^ DCs are considered to be the most potent cross-presenting subset of antigens including proteins, antibody-bound-, cell-associated, and other types of antigens *in vivo* and *ex vivo* (50–55). The explanations for the superior cross-presentation ability of CD8α^+^ DCs include lower degradation of antigen in endosomes by ROS production (56), more efficient transfer of exogenous antigens into the cytosol (57), and higher expression of components that are associated with MHCI processing pathway (55). Emerging data, however, suggest that the cross-presenting ability of each DC subset is tuned by and dependent on factors such as DC location and activation status, the type of antigen, and local inflammatory signals (58). Indeed, the main DC subset responsible for cross-presentation in lung, intestine and skin is the migratory CD103^+^ DCs (59, 60). Although CD8α^−^ DCs are generally considered to be the most potent MHCII antigen presenting subset to CD4^+^ T cells, it has been shown that CD8α^−^ DCs can efficiently cross-present antibody-bound antigen, antigens from *Salmonella typhimurium* and S. *cerevisiae*, or antigen in the presence of saponin adjuvants (61–65). CD8α^−^ DCs have been shown to cross-present antibody-bound antigen efficiently after activation of Fcγ-receptors (66), but a more recent study showed that complement factor C1q plays a dominant role in antibody-bound antigen uptake and cross-presentation in DCs (67). Although, some studies have shown the ability of pDCs to cross-present *in vitro* or *ex vivo* (34, 68, 69), their role in cross-presentation *in vivo* seems lacking during viral infections despite the fact that they are known for their ability in producing large amounts of type I interferons (70, 71). However, a recent study showed that upon TLR ligand activation, mitochrondial ROS production is increased independently of NOX2 in pDCs (72). Increased ROS production resulted in high endosomal pH, antigen protection from endosomal degradation, and induced export to the cytosol, ultimately leading to enhanced antigen cross-presentation and CD8^+^ T cell priming.

In human, the cDC subset in blood can roughly be divided into BDCA1^+^ (CD1c^+^) and BDCA3^+^ (CD141^+^) DCs (73). The BDCA1^+^ and BDCA3^+^ subsets are proposed as the human counterparts of murine CD8α^−^ and CD8α^+^ DCs, respectively. It has been shown that BDCA1^+^ DCs are capable of cross-presentation of extracellular antigen (74). Upon activation with TLR ligands, BDCA1^+^ DCs showed similar efficiency in cross-presentation compared to BDCA3^+^ DCs (75). A recent study showed that *in vivo* generated monocyte-derived DCs (moDCs) and monocyte-derived macrophages can both cross-present efficiently in a vacuolar-dependent pathway (76). In contrast to murine pDCs, the human counterpart has been reported to cross-present soluble, cell-associated antigen efficiently (77). However, recent work by the group of Ginhoux has identified a pre-DC subset that bears the classical pDC markers, including CD123, CD303, and CD304 (78). This pre-DC subset can be distinguished from the classical pDCs by additional markers, such as CD33, CX3CR1, CD2, CD5, and CD327. Importantly, they showed that only pre-DCs could induce CD4^+^ T cell proliferation and IL-12 production compared to classical pDCs. These data imply that the antigen presenting ability of pDCs might be a result of “contaminating” pre-DCs. Whether these pre-DCs can also cross-present to CD8^+^ T cells is currently unknown. It will be important to use additional markers to isolate pure pDC subset for future analysis of their antigen presenting capacity.

So far, most of the aforementioned studies investigating the molecular mechanisms of antigen cross-presentation make use of murine DC model systems and require confirmation in the human DC setting. Nevertheless, it seems that choosing specific antigen targeting routes can determine the outcome of DC cross-presentation efficiency of different subsets. Deciphering the molecular mechanisms of cross-presentation in the different DC subtypes in mice and human is needed for the optimal design of therapeutic vaccines.

## Clinically Relevant Adjuvants and Antigen Cross-Presentation

During the last years, many groups have been developing adjuvants that facilitate uptake by APCs, protect antigens against degradation and stimulate strong immune memory responses (79). Here, we will focus on new insights in the mode of action of clinically relevant adjuvants on antigen cross-presentation by DCs and subsequent induction of cellular immunity. Many studies analysing adjuvants show an enhancement of CD8^+^ T cells, but most studies do not differentiate between enhanced antigen cross-presentation by DCs or enhanced DC maturation, e.g., expression of co-stimulatory molecules and cytokines. Therefore, we will elaborate on those studies that describe the mechanisms of cross-presentation induced by adjuvants, including the involvement of the cytosolic and vacuolar pathway of cross-presentation in DCs. In addition, we will focus on clinically relevant adjuvants, including aluminum-based nanoparticles, saponin-based adjuvants (including ISCOMs), and TLR ligands.

### Aluminum-Based Nanoparticles

Aluminum salts are the most widely applied adjuvants in human vaccines and it is firmly established that they are safe and well-tolerated. Aluminum oxyhydroxide [AlO(OH)] is a positively charged vaccine carrier that strongly absorbs negatively charged antigens (80, 81). Its mechanisms of action include antigen retention and local inflammation via activation of the NLRP3. Either direct phagocytosis of the adjuvant or phagocytosis of stressed or dying cells that contain the aluminum salts and subsequent release of damage associated molecular patterns are able to activate the NLRP3 inflammasome (82). Aluminum adjuvants induce the production of IL-1β and IL-18 by DCs and a strong default Th2 differentiation promoting the production of antibodies (83). Therefore, current aluminum-based adjuvants exhibit a very limited potency to induce a cellular Th1 immune response as compared to other adjuvants (84).

**Figure 1:**
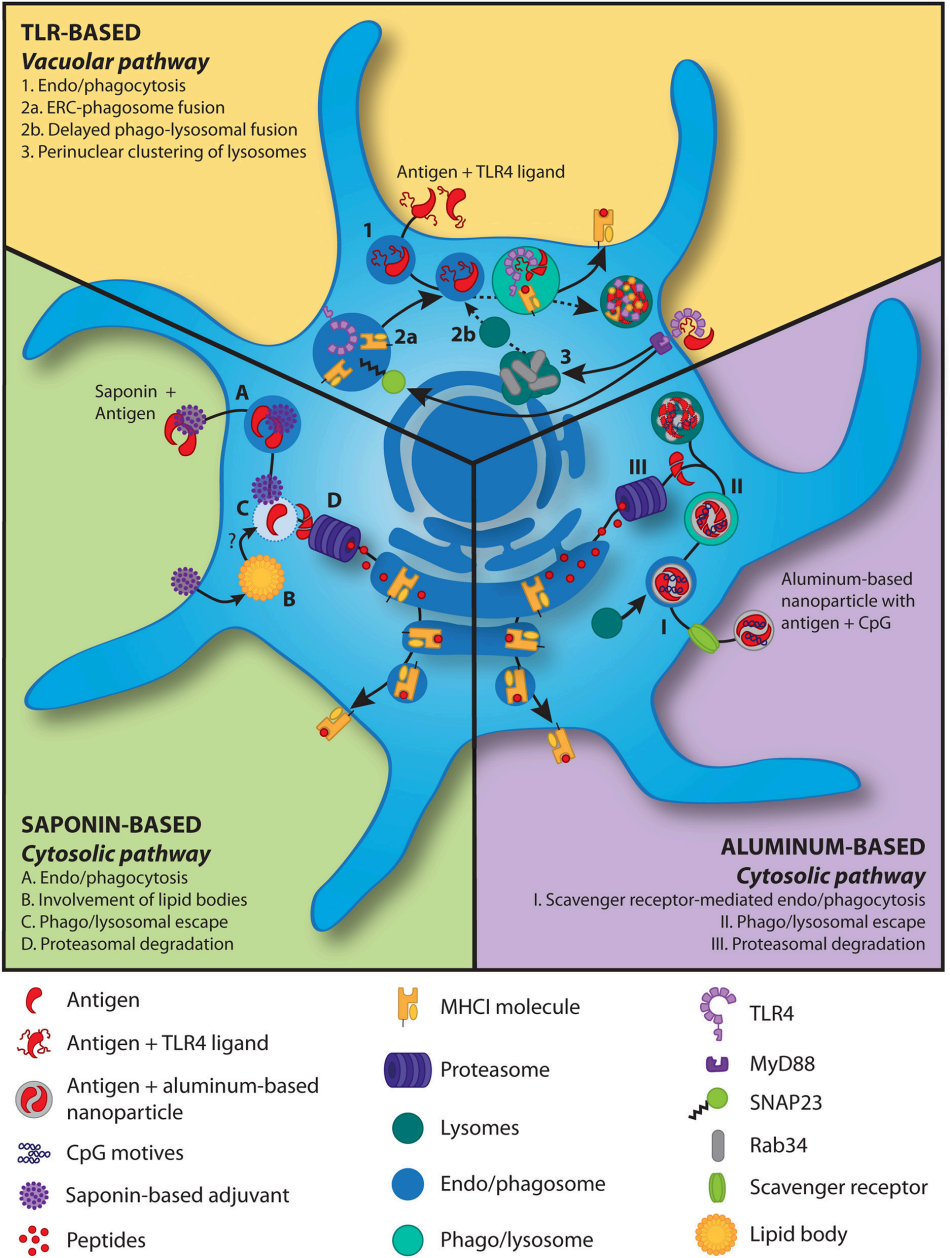
Models for antigen cross-presentation mechanisms induced by adjuvants in DCs. *TLR-based adjuvants*: In the presence of TLR triggering, antigen is taken up by the DCs and delivered to phago/lysosomes (1). The MHCI molecules and TLR4 within the endosomal recycling compartment are shuttled into the phago/lysosome (2a) following TLR4 signaling induced phosphorylation of SNAP23 (85). TLR4 signaling further induces perinuclear clustering (3) of lysosomes in a Rab34-dependent manner (86), resulting in delayed (dashed line) phago-lysosomal fusion (2b). The latter slows down antigen degradation and thereby increases cross-presentation. *Saponin-based adjuvants*: Saponins, alone or in phospholipid and cholesterol particles, in combination with antigens are phagocytosed (A). The saponins induce lipid bodies (B) and increase cytosolic translocation of the antigen (C) and subsequent proteasome-dependent cross-presentation (D) (65, 87) via the cytosolic pathway. Lipid bodies play an unknown but crucial role in this process (B) (65). *Aluminum-based nanoparticles*: An aluminum-based nanoparticle loaded with antigen and the TLR9 ligand CpG is taken up via endocytosis, which is largely mediated through the scavenger receptor A (I) (80). After lysosomal fusion with the endosome, nanoparticle-mediated rupture of the vesicular membrane gains antigens access to the cytosol (II) and after proteasomal degradation (III) are cross-presented via the cytosolic pathway.

Interestingly, Jiang et al. transformed the micrometer-sized aggregates of AlO(OH) adjuvant into nano-sized vaccine carriers by shielding its positive charge with a polyethylene glycol (PEG)-containing polymer (80). The resulting nanoparticles could be readily co-loaded with both antigen and the TLR ligand CpG without affecting size or Zeta-potential of the particles and these particles were effectively internalized by murine APCs. Using endocytic pathway inhibitors, they showed that internalization is highly dependent on scavenger receptor A-mediated endocytosis (Illustrated in Figure 1). Confocal microscopy revealed localization of the nanoparticles within the lysosomes as well as in the cytosol, indicating lysosomal escape. The cytosolic delivery of the nanoparticles is possibly caused by AlO(OH) induced destabilization of lysosomes as described previously by others (88). Most importantly, Jiang et al. showed that cytosolic delivery of the nanoparticles containing OVA protein effectively promotes cross-presentation by DCs compared to free OVA protein, as measured by a monoclonal antibody specifically detecting MHCI/OVA peptide complexes. Strikingly, the presence of CpG in the nanoparticle further enhanced the level of antigen cross-presentation by DCs. Further analysis revealed that brefeldin A, which inhibits protein transport from the ER to Golgi, and MG-132, which inhibits the proteasome, reduced DC cross-presentation, while the cysteine protease inhibitor leupeptin did not. These data are thus consistent with the cytosolic route being the dominant cross-presentation pathway activated by the nanoparticle. Interestingly, while the size and positive charge at neutral pH of AlO(OH) in the traditional vaccine prevented its targeting to lymph nodes, AlO(OH) packed into nanoparticles of < 90 nm in diameter efficiently reached lymph node APCs *in vivo*. Especially, nanoparticles loaded with CpG were able to expand and mature DCs in the lymph nodes and induced production of TNF-α and IL-12p70. Moreover, the presence of CpG in the AlO(OH) nanoparticles was necessary for the effective induction of both IgG1 and IgG2 responses as well as strong CD8^+^ T cell response and delayed growth of B16 melanoma tumors. Control vaccination with CpG and OVA antigen without the AlO(OH) nanoparticles was much less effective. In conclusion, AlO(OH) nanoparticles in combination with CpG is a very potent and promising adjuvant combination for the induction of cellular immune responses.

Two other studies using AlO(OH) adjuvant packed into nanoparticles confirm this is a promising strategy to promote cross-presentation and/or cross-priming. Dong et al. synthesized AlO(OH) nanoparticles containing a polyethyleneimine (PEI) modification to increase antigen loading capacity (89). Particles were successfully loaded with tumor autophagosome derived proteins that are potentially enriched for tumor associated antigens. Zhao et al. created Al_2_O_3_ nanoparticles containing the Vx3 ubiquitin binding protein to enrich for ubiquitinated proteins present in tumor lysates, also to potentially enrich for tumor associated antigens (90).

Thus, the application of aluminum-based adjuvants showed that the use of aluminum salts can be improved by using nano-sized particles, especially in combination with TLR ligands, and that cross-presentation by DCs can be enhanced. The AS04 adjuvant is clinically approved, and is a combination of MPL and aluminum salt (Table 1). AS04 has shown to be very potent and the aluminum hydroxide is able to prolong the MPL induced cytokine response. The fact that this vaccine is successfully used in the clinic demonstrates that aluminum can be a useful carrier of other immunostimulatory molecules and that combining adjuvants is a promising strategy for the induction of strong cellular immune responses.

### Saponin-Based Adjuvants

Saponins are triterpene glycosides derived from the bark of the South American soapbark tree, *Quillaja saponaria*. Dalsgaard has obtained a heterogeneous mixture of soluble *Quillaja*-derived saponins, Quil-A®, which has been commercialized and used in veterinary studies showing humoral and cellular immunity (91, 92). Further, purification of this mixture led to the identification of 10 fractions containing adjuvant activity, including QS-21 (93). Since QS-21 showed the least hemolytic effect compared to the other fractions, it was extensively investigated as an adjuvant. QS-21 can induce a robust antibody and cell-mediated immune response activating both Th1 and CD8^+^ T cells (94). QS-21 has been proposed to exert its immunomodulatory effects by acting on different cell types *in vivo* [reviewed in (95)]. One study has shown that QS-21 can activate NLRP3 inflammasomes to induce IL-1β and IL-18 production in murine DCs (96). However, NLRP3-deficient mice showed higher levels of Th1 and Th2 antigen-specific T cell responses and increased IgG1 and IgG2c in the presence of QS-21, thus suggesting a more complex regulatory role for NLRP3. In human moDCs QS-21 has been reported to facilitate non-receptor-mediated uptake of exogenous antigen in a cholesterol-dependent manner (87). After endocytosis of antigen and QS-21, both are transported to the lysosomes where QS-21 causes lysosomal destabilization, followed by antigen release in the cytosol for further processing and cross-presentation (Illustrated in Figure 1). Moreover, they showed that cell activation depends on the activity of Syk kinase and cathepsin B, since Syk knockdown blocked NF-κB activation and cytokine production (IL-6 and TNF) in moDCs and shRNA-mediated knockdown of cathepsin B strongly decreased the expression of both TNF and IL-6 mRNAs. Moreover, cathepsin B-deficient mice showed lower cytokine (IL-2, TNF, and IFN-γ)-producing antigen-specific T cells. Neither for human nor for murine DCs has the mode of action of QS-21 on DC cross-presentation efficiency been investigated in detail.

When Quillaia saponins are admixed with cholesterol and phospholipid they spontaneously form open cage particles with a diameter of ~40 nm, termed immune stimulating complexes (ISCOMs) (97). Due to the interaction of saponin with cholesterol, saponin is thought to be protected from hydrolysis and thereby stabilizing the adjuvant (98). Moreover, toxic side effects are greatly reduced since saponin interaction with membranes is decreased (99), while induction of antigen-specific T cell responses, prolonged antibody responses, and a balanced Th1/Th2 immunity are equal or even more potent (100, 101). In this review we will address the different saponin formulations as saponin-based adjuvants (SBAs).

Duewell et al. showed that SBA vaccines injected subcutaneously in mice resulted in the recruitment and activation of innate and adaptive immune cells in vaccine site-draining lymph nodes. They showed efficient uptake of antigen in DCs, induction of DC maturation, and IL-12 production *in vivo* (102). Moreover, they showed enhanced antigen cross-priming by CD8α^+^ murine DCs relative to antigen alone, measured by induction of T cell proliferation, as well as protective anti-tumor immunity. The SBA vaccine induced activation and MHCI cross-priming by DCs in murine draining lymph nodes in a TLR-signaling adapter MyD88-independent manner (64). On the contrary, CD8^+^ T cell-priming, NK cell activation, and potent antitumor activity in a prophylactic tumor challenge model *in vivo* were MyD88-dependent, suggesting a more downstream role of MyD88. They further showed that SBA induced efficient cross-priming by both CD8α^−^ CD205^+^ DCs as well as CD8α^+^ CD205^+^ DCs in draining lymph nodes 24 hours after vaccination. Surprisingly, murine splenic CD4^+^ DCs were more efficient than CD8α^+^ DCs at cross-priming soluble antigen formulated with SBA. Studies using another SBA formulation called Matrix-M^TM^, which consists of two individually formed particles, Matrix-A and Matrix-C, together with cholesterol and phospholipid, also showed an increase in CD8^+^ and CD4^+^ T cell responses and 100% protection in a lethal viral challenge murine model (103). However, the precise mechanism how T cell induction was achieved was not investigated.

Two recent papers provide more insight in the mechanism of SBA induced cross-presentation by DCs. They demonstrated that saponin fraction C alone or formulated as an SBA can both induce an unprecedented level of DC cross-presentation in murine GM-CSF generated DCs *in vitro*, as shown by activation of the co-stimulation independent B3Z reporter T-cell line (47, 65). Moreover, SBA encounter did not change levels of CD80 or CD86 on *in vitro* cultured murine DCs. They further demonstrated that SBA predominantly act by inducing cross-presentation in the monocytic CD11b^+^ DC subset *in vitro* and *in vivo*, a population distinct from the well-described CD8α^+^ cross-presenting DCs. The presence of SBA increased cytosolic translocation of antigen, resulting in proteasome-dependent cross-presentation. Strikingly, specifically in this monocytic CD11b^+^ DC subset, SBA enhanced DC cross-presentation by lipid body induction. Both pharmaceutical and genetic interference with lipid body formation inhibited the SBA-induced cross-presentation in these DCs *in vitro* and *in vivo* (Illustrated in Figure 1).

Human moDC studies have shown that SBA induced efficient cross-presentation of the cancer testis antigen NY-ESO-1 based on IFN-γ production by CD8^+^ T cells (101). Interestingly, NY-ESO-1/SBA cross-presentation was studied for three distinct HLA-restricted epitopes. Independent of whether NY-ESO-1 is delivered in combination with SBA as two separate entities or formulated into one particle (ISCOMATRIX), the generation of two epitopes (HLA-A2, HLA-Cw3) was proteasome independent while the generation of the third epitope was highly proteasome dependent, as was the processing of the melanoma-differentiation antigen Melan-A when combined with SBA. Further analysis uncovered that cytosolic tripeptidyl peptidase II (TPPII) was involved in the generation of the HLA-A2, HLA-Cw3 epitopes of the NY-ESO-1/SBA vaccine. In line with this finding, they showed rapid antigen translocation from lysosomes into the cytosol in the presence of SBA. Thus, SBA vaccines are compatible with both cytosolic TPPII and the proteasome to generate immunogenic epitopes for MHCI antigen cross-presentation. In a follow-up study they showed that *in vitro* generated moDCs and freshly isolated CD1c^+^ DCs from blood could both cross-present NY-ESO-1 and Melan-A epitopes (104). However, when the antigen was limited, moDCs were more efficient than CD1c^+^ DCs in cross-presentation *in vitro*. In addition, under these conditions physically incorporating the antigen into SBA (ISCOMATRIX) was superior compared to separate administration of antigen and adjuvant to CD1c^+^ DCs. In conclusion, also in human DCs, SBAs can efficiently induce DC cross-presentation and different epitopes from the same protein can be processed by different pathways in DCs.

Currently, only the saponin QS-21 is approved for use in formulation with MPL as AS01 adjuvant in a human vaccine against malaria (Table 1). Furthermore, QS-21 has been added as adjuvant to a recombinant retroviral subunit vaccine against feline leukemia virus (105) in cats. In the human setting, SBAs in combination with NY-ESO-1 protein have now also been used in human clinical trials in patients with NY-ESO-1^+^ tumors, generating high-titer antibody responses, and strong CD8^+^ and CD4^+^ T cell responses (106). To further extend the clinical application of SBAs, it will be important to fully understand the mode of actions of the adjuvant on cross-presentation by different DC subsets, including the role of lipid body induction. In addition, defining saponin adjuvant antigen formulations showing limited side effects while inducing maximal antigen cross-presentation capacity should further pave the way for their clinical application.

### TLR Ligands

TLR ligands are well-known for their ability to induce DC maturation resulting in expression of co-stimulatory molecules and pro-inflammatory cytokines. The capacity to induce potent cellular immunity makes them a powerful addition to the armamentarium for cancer vaccinations. Interestingly, recent studies show that TLR ligands can also have direct effects on cross-presentation by DCs, making TLR ligands even more attractive for use in cancer vaccines. Upon TLR4-induced DC maturation, cross-presentation is first enhanced and followed by down-modulation of antigen internalization and cytosolic delivery (107). The two following studies focus on the first hours following TLR4 activation, in which the cross-presentation capacity is increased (85, 86).

Nair-Gupta et al. described a new pathway, in which TLR signaling, especially TLR4 triggering, can lead to increased cross-presentation by murine DCs (85). They showed that Escherichia coli expressing OVA protein (*E. coli*-OVA) is able to induce cross-priming of CD8^+^ T cells by wildtype DCs, but not by Trif^−/−^MyD88^−/−^ DCs. Trif^−/−^MyD88^−/−^ DCs could induce CD8^+^ T cell priming when provided with the pre-processed SIINFEKL epitope, thereby excluding a general inability to activate T cells. Confocal microscopy analysis showed the selective accumulation of MHCI molecules within the LAMP1^+^ phagosomes also carrying the TLR4 ligand. These MHCI molecules were shown to be derived from the perinuclear Rab11a^+^ vesicle-associated membrane protein (VAMP)3/cellubrevin^+^ and VAMP8/endobrevin^+^ endosomal recycling compartment (ERC) which contains large amounts of MHCI. Silencing Rab11a dissolved the existence of the perinuclear reserves of MHCI and diminished TLR-mediated cross-presentation. Of note, these Rab11a^+^ MHCI^+^ pools are predominantly found in the CD8α^+^ DCs, suggesting that the existence of MHCI pools contributes to their strong cross-presentation capacity. Trafficking of MHCI from the ERC to the phagosome is, however, Rab11a independent but controlled by TLR4 induced IKK2-dependent phosphorylation of SNAP23. In conclusion, TLR signaling, especially via TLR4 leads to phosphorylation of SNAP23 and SNAP23-mediated trafficking of the perinuclear MHCI pools from the ERC to the LAMP1^+^ TLR ligand^+^ phagosomes (Illustrated in Figure 1). Alloatti et al. uncovered another mechanism how LPS treatment of DCs results in improved cross-presentation of both soluble and bead-bound OVA protein as well as proliferation and activation of antigen specific CD8^+^ T cells *in vitro* and *in vivo* (86). By single organelle-based flow cytometry they showed that upon LPS stimulation, phagosomes contained more OVA protein and expressed less LAMP1, indicating less antigen degradation and lower levels of phago-lysosomal fusion, respectively. This effect was completely dependent on TLR4. Liquid chromatography-tandem mass spectrometry analysis of phagosomal proteins of both resting DCs and LPS stimulated DCs showed that phagosomes of resting DCs were highly enriched for the majority of lysosomal hydrolases, consistent with the LPS induced reduction in phago-lysosomal fusion. Moreover, LPS induced perinuclear clustering of LAMP1^+^ lysosomes in maturing DCs, while broad peripheral distribution was observed in unstimulated DCs. This same perinuclear clustering was previously seen by Nair-Gupta et al. upon TLR stimulation (85). The perinuclear accumulation of lysosomes delayed phagosome maturation and phago-lysosomal fusion, resulting in improved cross-presentation, which was controlled by the GTPase Rab34 (Illustrated in Figure 1). Rab34 has been previously linked to cross-presentation efficiency (108). Interestingly, TLR7 and TLR9 activating ligands were able to show similar effects, but to a lower extent. Since antigen degradation is not mediated through the proteasome and loading of MHCI molecules with antigen does not happen in the ER but in the phago/lysosome, we believe the vacuolar pathway is followed.

TLR9 ligand CpG has potent immunostimulatory adjuvant activity and preferentially induces Th1 responses and tumor-specific CD8^+^ T cells (109, 110). As TLR9 is located intracellularly, CpG needs to be internalized to exert its immunomodulatory effect. Consistent with the aforementioned findings, the cross-priming ability of murine DCs was shown to be dependent on the colocalization of antigen and TLR9 ligand in the same endocytic compartment within DCs (111, 112). Indeed, the failure or success of CpG as an adjuvant in the tumor setting was dependent on the timing of CpG relative to the release of tumor antigen following ablation (111). Similarly, combining TLR ligand and antigen in the same vaccine particle is more potent compared to separate administration (112). Thus, addition of a TLR ligand as an adjuvant to a vaccine is a promising treatment strategy to induce both enhanced cross-presentation and cross-priming by DCs.

In summary, since their discovery a lot of knowledge has been acquired regarding the mode of action of TLRs and their ligands, including their role in antigen cross-presentation. Many TLR ligands have now also been tested as adjuvants for therapeutic cancer vaccines in clinical trials. However, only MPL has been approved as a purified TLR ligand for clinical use in several adjuvants (Table 1) (113). It will be interesting to test MPL as well as other TLR ligands in clinical development for their capacity to induce antigen cross-presenting in human DC subsets for future clinical application.

## Future Perspectives

For vaccines aiming to induce cell-mediated immunity such as cancer vaccines, it is important they stimulate both antigen cross-presentation by DCs and DC maturation to initiate an optimal CD8^+^ T cells response. The “ideal” adjuvant thus combines both these characteristics and is able to prolong antigen exposure to the immune system. SBAs stand out to enhance DC cross-presentation, but are relatively poor in immune activation. Therefore, additional DC activation by e.g., TLR ligands is crucial. Moreover, combination of multiple PRR agonists can induce synergistic effects on DC activation (114). Furthermore, activating both the vacuolar and cytosolic pathway might be beneficial to enhance DC cross-presentation. To achieve prolonged antigen exposure another type of adjuvant formulation might be required. Based on pre-clinical as well clinical data, a picture is emerging that an optimal vaccine adjuvant may actually require a combination of adjuvants rather than a single adjuvant entity. The clinically approved vaccines adjuvants AS01, AS02, and AS04 show that a combination of different adjuvants, especially TLR ligands combined with other adjuvant(s) such as saponins or alum, can be both potent and safe to use in the clinic.

An important aspect to consider when choosing an adjuvant is that different DC subsets show differential cross-presentation efficiencies, which makes it important to study the response in subsets and potentially even to specifically target the most effective subsets. Targeting antigens directly to DCs using antibodies is explored for better antigen uptake, DC activation and thereby T cell-mediated immunity. Moreover, directly targeting specific DC subsets or receptors that allow strong cross-presentation can further enhance immune responses. Many studies targeting C-type lectin receptors on DCs including DEC205, DC-SIGN, and DNGR1 (Clec9A) showed efficient antigen-specific CD8^+^ T cell responses (115). A potential drawback of (too) specific DC targeting is that *in vivo* the different DC subsets are known to work in concert and that antigen presentation by different DC subsets during the course of an immune response may be important to unleash a powerful immune response. Also vaccines with a different design, that are beyond the scope of this review, showed promising results, including the work of Sahin et al. (116, 117). Vaccines consisting of RNA encoding tumor antigen derived epitopes and containing immunostimulatory motifs were delivered by nano-sized lipoplexes that preferentially target and activate DCs in the spleen and have already been tested in a few patients. It is important to realize that so far, most of the studies looking into the potency and mode of action of adjuvants use murine DCs and hardly differentiate between different DC subsets. Extrapolation of the murine data on adjuvants to human DCs and preferentially also DC subsets will be important for future clinical application. It may be especially rewarding to test adjuvants in clinical development for their capacity to induce antigen cross-presenting by human DCs to select for adjuvants inducing T cell-mediated immunity. In conclusion, many aspects, from choosing the antigen, targeting specific DC subsets, activating DCs via PRR signaling, to stimulating efficient DC cross-presentation, need to be considered when choosing a vaccine and adjuvant. Understanding the underlying mechanisms will boost the development of next generation vaccines for clinical application.

## Author Contributions

NH, LH, and GA wrote the manuscript. TR reviewed the manuscript and made the figure illustration.

### Conflict of Interest Statement

The authors declare that the research was conducted in the absence of any commercial or financial relationships that could be construed as a potential conflict of interest.
